# Crosstalk between protein kinases AKT and ERK1/2 in human lung tumor-derived cell models

**DOI:** 10.3389/fonc.2022.1045521

**Published:** 2023-01-04

**Authors:** Aurimas Stulpinas, Matas Sereika, Aida Vitkeviciene, Ausra Imbrasaite, Natalija Krestnikova, Audrone V. Kalvelyte

**Affiliations:** Department of Molecular Cell Biology, Institute of Biochemistry, Life Sciences Center, Vilnius University, Vilnius, Lithuania

**Keywords:** cancer cell, cell signaling, ERK (extracellular signal-regulated kinase), AKT, kinase inhibitors, targeted drugs, cellular resistance, lung cancer

## Abstract

There is no doubt that cell signaling manipulation is a key strategy for anticancer therapy. Furthermore, cell state determines drug response. Thus, establishing the relationship between cell state and therapeutic sensitivity is essential for the development of cancer therapies. In the era of personalized medicine, the use of patient-derived ex vivo cell models is a promising approach in the translation of key research findings into clinics. Here, we were focused on the non-oncogene dependencies of cell resistance to anticancer treatments. Signaling-related mechanisms of response to inhibitors of MEK/ERK and PI3K/AKT pathways (regulators of key cellular functions) were investigated using a panel of patients’ lung tumor-derived cell lines with various stemness- and EMT-related markers, varying degrees of ERK1/2 and AKT phosphorylation, and response to anticancer treatment. The study of interactions between kinases was the goal of our research. Although MEK/ERK and PI3K/AKT interactions are thought to be cell line-specific, where oncogenic mutations have a decisive role, we demonstrated negative feedback loops between MEK/ERK and PI3K/AKT signaling pathways in all cell lines studied, regardless of genotype and phenotype differences. Our work showed that various and distinct inhibitors of ERK signaling – selumetinib, trametinib, and SCH772984 – increased AKT phosphorylation, and conversely, inhibitors of AKT – capivasertib, idelalisib, and AKT inhibitor VIII – increased ERK phosphorylation in both control and cisplatin-treated cells. Interaction between kinases, however, was dependent on cellular state. The feedback between ERK and AKT was attenuated by the focal adhesion kinase inhibitor PF573228, and in cells grown in suspension, showing the possible role of extracellular contacts in the regulation of crosstalk between kinases. Moreover, studies have shown that the interplay between MEK/ERK and PI3K/AKT signaling pathways may be dependent on the strength of the chemotherapeutic stimulus. The study highlights the importance of spatial location of the cells and the strength of the treatment during anticancer therapy.

## 1 Introduction

Nowadays modern trends in cancer biology and cancer treatment are designed to address the problem of drug resistance. They encompass various aspects of the phenomenon, such as resistance to conventional and targeted drugs, hormone therapy, and immune oncology agents in various types of tumors. Various causes, modes, and mechanisms of resistance, ranging from genetic to phenotypic, have been described in many reviews, including ours (1). There is no doubt that, in addition to genetic changes, the acquisition of non-genetic causes of resistance determines the outcome of cancer treatment. The mechanisms of resistance to anticancer drugs are diverse. Genetic alterations in cancer cells or external factors in the tumor microenvironment may lead to decreased drug uptake, accelerated drug clearance, adaptive cellular metabolism, or altered drug-target interactions that promote drug resistance, etc. (2–5).

Lung cancer is one of the most common types of malignancy and the leading cause of cancer death worldwide (6). The majority of all lung cancers are non-small cell lung cancer (NSCLC). Somatic alterations in many different genes encoding regulatory molecules that are responsible for oncogenic transformation include mutations, insertions, gene amplifications, etc., and are known to cause lung cancer. Among them are mutations in the *EGFR, FGFR, BRAF, AKT1*, and *KRAS* genes, and rearrangements in *ROS1, NTRK1, ALK*, and *RET* are common (7). Most of the targeted drugs currently used in the treatment of lung cancer are specific for oncoproteins encoded by these somatically corrupted genes, and cancer treatment is based on tumor genome profiling (3).

Current treatment of NSCLC includes surgery, chemotherapy, radiotherapy, and targeted therapy. Chemotherapy continues to be one of the mainstays of treatment for many patients with advanced non-small cell lung cancer and involves conventional cytotoxic chemotherapeutic drugs (cisplatin, oxaliplatin, gemcitabine, paclitaxel, docetaxel, pemetrexed, vinorelbine). Up to now, platinum and its derivatives are still the most widely used chemotherapeutic drugs for lung tumors. As in other cases, however, these chemotherapeutic drugs generally face the problem of drug resistance, diminishing the therapeutic efficacy (8, 9). Accumulating evidence indicates that signaling pathways that are involved in the regulation of cell proliferation and survival, such as PI3K/AKT and MAPK, contribute to cisplatin resistance (10, 11). Extracellular signal regulated kinases (ERK1 and ERK2) and their upstream activators MAPK-ERK kinases (MEK1 and MEK2) belong to the MAPK signaling pathway.

Targeted therapy already used in NSCLC treatment involves targeted drugs, small-molecule inhibitors of cellular signaling components (gefitinib, erlotinib, crizotinib, ceritinib, alectinib, osimertinib, afatinib, lorlatinib, selpercatinib), and antibodies (atezolizumab, pembrolizumab, bevacizumab, necitumumab, ramucirumab, nivolumab) (12). Several new targeted drugs (among them entrectinib, tepotinib, mobocertinib, selpercatinib, and sotorasib) are in clinical trials, some pending accelerated approval (13). These medications are often used in combinations since targeted drug monotherapy results in resistance and cancer recurrence (2).

There is no doubt that the introduction of targeted therapies has fundamentally changed the treatment of cancer. Despite advances in cancer treatment, including lung cancer, however, it should be noted that emerging drug resistance and cancer recurrence are observed in most cases. As in other cases, only transient improvements in the treatment of lung cancer are observed (2, 14). In that regard, it is therefore important to re-evaluate existing treatment and drug resistance strategies for different cancer cell options and to provide new treatments for different cases.

It is known that cancerous signaling induced by the abovementioned genes, mutated in lung cancer, includes activation of ERK and AKT protein kinases. Accordingly, targeted therapy directed to these two majors signaling pathways has been newly proposed as a promising, alternative treatment for NSCLC (7). ERK and AKT transmit external and internal signals from growth factors or intercellular contacts and adhesion signals to reprogram transcription. They regulate essential cellular functions, such as proliferation, survival, growth, metabolism, migration, and differentiation (15–17). ERK and AKT signaling pathway molecules are often activated by a variety of different oncogenic mutations that are found in multiple human cancers. These signaling pathways are considered to be the main transducers of oncogenic signals and thereby participate in cancer progression. In some cases, gene mutations in these pathways have been identified (18–22). ERK and AKT also mediate the cellular response to anticancer drugs, and deregulation of these kinase signaling is often associated with resistance to therapy. ERK and AKT kinases are, therefore, attractive candidates for improving the efficacy of targeted and conventional chemotherapy.

In this study, ERK and AKT kinase-targeted drugs and their combinations with conventional chemotherapeutics were tested on lung adenocarcinoma cell line A549 and *ex vivo* lung cancer patient-derived heterogeneous cell line sets as a model system. Studies are focused on cell-phenotype-driven cancer resistance to therapy. The effectiveness of conventional and targeted drugs selected to inhibit the intracellular signal-transducing protein kinases was tested by using different exposure and *in vitro* cell culture conditions.

Inhibitors of the ERK and AKT signaling pathways from different clinical development phases or already approved by the U.S. Food and Drug Administration (FDA) were used in the study: selumetinib, trametinib, SCH779248, idelalisib, and capivasertib. MAPK-targeted treatment has been successfully applied in melanoma and neurofibromatosis although they are not yet approved for the treatment of lung cancer (23–26).

Increasing evidence indicates the non-oncogenic dependencies of drug resistance. The diversity of non-genetic mechanisms is determined by changes in cell status, epithelial-mesenchymal transitions, and differentiation, depending on the tumor microenvironment, and is characterized by differences in epigenetic, transcription, signal transduction, metabolism, and DNA damage responses mechanisms (14, 27–31). Understanding non-mutational mechanisms of cell resistance, as well as new models for drug sensitivity testing, must be important in finding new avenues for the treatment of cancer.

Compensatory activation of signaling pathways, as in other cancers, is known to be one of the major obstacles to targeted therapy in non-small cell lung cancer (32, 33). This study aimed to assess the phenomenon of the feedback loop between MEK/ERK and PI3K/AKT in various cells of lung tumor origin and to evaluate its possible dependence on extracellular contacts and the extent of treatment (drug dosing).

## 2 Materials and methods

### 2.1 Cell culture

Human non-small cell lung carcinoma A549 cells were purchased from CLS (Cell Lines Service GmbH, Eppelheim, Germany). Human primary lung cancer cell lines were established from surgical material (Regional bioethical approval no. 158200-18/5-1024-537) as previously described (34). Briefly, lung tumor specimens from 9 patients were minced, washed, and enzymatically digested. The digested tissue was filtered through a cell strainer and transferred into a cell culture flask with Iscove’s modified Dulbecco’s medium (IMDM, Gibco, # 21980032) supplemented with 10% FBS (Gibco, #10500064) and antibiotics (Gibco, #15240062). Cells were grown at various densities and under different culture conditions. Cells of passage number 10–30 were used in our experiments. Agitation experiments were performed in an environmental orbital shaker-incubator (ES-20/60, Biosan, Riga, Latvia) at 37°C in a CO_2_-independent medium (Gibco, #18045088) supplemented with glutamine, 10% FBS and antibiotics.

### 2.2 Cell morphology

Cell morphology was observed, and the micrographs of the cells were obtained with an EVOS FL microscope (Thermo Scientific) using 10x objective with phase contrast for brightfield. Cell staining was performed with crystal violet (0.1% in 20% ethanol) dye. Scale bars 400 µm (magnification 10x).

### 2.3 Gene expression analysis by RT-qPCR

Total RNA was purified from cells using “TRIzol Reagent” (Thermo Fisher Scientific, Waltham, MA, USA) according to the manufacturer’s instructions. Contaminating genomic DNA was removed from RNA samples using “DNase I, RNase-free” (Thermo Fisher Scientific). cDNA was synthesized using “Maxima First Strand cDNA Synthesis Kit for RT-qPCR” (Thermo Fisher Scientific), and qPCR was performed using “Maxima SYBR Green/ROX qPCR Master Mix (2X)” (Thermo Fisher Scientific) on the RotorGene 6000 system (Corbett Life Science, QIAGEN) according to the manufacturer’s instructions. Gene-specific primer sequences (Metabion international AG, Planegg/Steinkirchen, Germany) are presented in **Table 1**. Relative gene expression was calculated using the ΔΔCt method. *GAPDH* was used as a “housekeeping” gene.

**Table 1:** Primers used for RT-qPCR analysis.

Gene	Forward and reverse primers
*ABCG2*	F: TATAGCTCAGATCATTGTCACAGTC R: GTTGGTCGTCAGGAAGAAGAG
*ALDH1*	F: GGGCAGCCATTTCTTCTCAC R: CTTCTTAGCCCGCTCAACAC
*E-cadh*	F: TGAGTGTCCCCCGGTATCTT R: GAATCATAAGGCGGGGCTGT
*FOXC2*	F: CGCCCGAGAAGAAGATCACC R: CGCTCTTGATCACCACCTTC
*FOXQ1*	F: ACGCTGGCGGAGATCAACGAG R: AGGTTGTGGCGCACGGAGTT
*GAPDH*	F: AGTCCCTGCCACACTCAG R: TACTTTATTGATGGTACATGACAAGG
*HOXA5*	F: TTTTGCGGTCGCTATCC R: CTGAGATCCATGCCATTGTAG
*MYC*	F: CAGCGACTCTGAGGAGGAAC R: GCTGTGAGGAGGTTTGCTGT
*N-cadh*	F: TGCGGTACAGTGTAACTGGG R: GAAACCGGGCTATCTGCTCG
*NANOG*	F: AGATGCCTCACACGGAGACT R: GTTTGCCTTTGGGACTGGTG
*OCT4*	F: CGAGAAGGATGTGGTCCGAG R: CAGAGGAAAGGACACTGGTC
*SOX2*	F: TGGACAGTTACGCGCACAT R: CGAGTAGGACATGCTGTAGGT
*SOX9*	F: GAGGAAGTCGGTGAAGAACG R: ATCGAAGGTCTCGATGTTGG
*SNAIL1*	F: CCAGACCCACTCAGATGTCAAGAA R: GGCAGAGGACACAGAACCAGAAAA
*SNAIL2*	F: GGCAAGGCGTTTTCCAGAC R: GCTCTGTTGCAGTGAGGGC
*SYK*	F: ACTTGGTCAGCGGGTGGAAT R: GGGTGCAAGTTCTGGCTCAT
*TWIST1*	F: CCTTCTCGGTCTGGAGGAT R: TCCTTCTCTGGAAACAATGACA
*TWIST2*	F: CGCAAGTGGAATTGGGATGC R: CGATGTCACTGCTGTCCCTT
*ZEB1*	F: GTTACCAGGGAGGAGCAGTGAAA R: GACAGCAGTGTCTTGTTGTTGTAGAAA
*ZEB2*	F: AGGAGCTGTCTCGCCTTG R: GGCAAAAGCATCTGGAGTTC

### 2.4 Inhibition and prevention of cell adhesion

To investigate the role of extracellular contacts, particularly the role of integrins, we maintained the cells in suspension for 24 hours. To prevent their adhesion to tissue culture plates, trypsin-detached cells in a CO_2_-independent medium were placed in non-treated cell culture flasks T-75 (Eppendorf Austria GmbH, Vienna, Austria, #0030711017) and constantly agitated at 90 rpm (orbit diameter 20 mm) for 24 hours in an orbital shaker-incubator. For further experiments, the suspension was divided into non-treated flasks T-25 and then exposed to inhibitors of selected kinases. After 6 hours, the cell suspension was fractionated into single-cell and aggregated cell fractions by natural sedimentation (1 hour in a vertical 15 mL tube at 37°C). Single cells from suspension were collected by centrifugation and lysed for protein analysis. Adherent cells grown in conventional cell culture-treated T-25 flasks with the same CO_2_-independent medium were used as controls.

For focal kinase (FAK) inhibition studies, cells were seeded into standard 6-well tissue culture-treated plates (Corning, New York, USA, #353046) for adhesion overnight. The FAK inhibitor PF573228 (Merck) (10 μM) followed by the ERK and AKT signaling pathway inhibitors was then added to the new medium for 6 hours. The cells were then lysed and the extracted proteins were analyzed by the Western blot method.

### 2.5 Inhibitory analysis

The number of cells prior to experimentation was ascertained by Bio-Rad TC20 automatic cell counter after trypsinization (TrypLE, Gibco, #12604013). Lung cancer-derived cells were treated with the chemotherapeutic drug cisplatin (cis-diammineplatinum(II) dichloride (Sigma-Aldrich)) for drug resistance studies. The following inhibitors of ERK and AKT signal pathways were used in this study: PI3K inhibitor idelalisib CAL-101 (10 µM; Cayman Chemical, Ann Arbor, MI, USA); Akt inhibitor VIII (10 µM, Merck) and capivasertib AZD5363 (10 µM, Cayman chemical); MEK1/2 inhibitors selumetinib AZD6244 (10 µM, Selleck Chemicals, Houston, TX, USA), trametinib (1 µM, Cayman chemical); ERK inhibitor SCH772984 (1 µM, Cayman chemical); FAK inhibitor PF573228 (10 µM, Merck.). Cell viability after the treatments was measured using the MTT (3-(4,5-dimethylthiazolyl-2)-2,5-diphenyltetrazolium bromide (from Sigma-Aldrich)) assay. "Relative cell viability" in the figures stands for the ratio of the measured values after the treatment with either the initial control (in cisplatin dose-response experiments) or the values from the treatment with cisplatin+DMSO alone (for combinational treatments). "Proliferation index" refers to the measured value after the treatment, normalized to the DMSO-treated control (72 hours). Water-insoluble MTT formazan was dissolved in ethanol and quantified spectrophotometrically reading absorbance at 570 nm in a Varioskan Flash plate reader.

### 2.6 Western blotting

For the protein phosphorylation analysis cells were lysed in lysis buffer (10 mM Tris-HCl buffer (pH 7.4) containing 50 mM NaCl, 5 mM EDTA, 50 mM NaF, 1% Triton X-100, 1 mM PMSF, 2 mM Na_3_VO_4_ and 20 µg/mL aprotinin). An equal amount of protein (Bradford assay) was run in SDS-PAGE. Proteins were transferred onto a PVDF membrane. After blocking with 5% non-fat milk powder in TBST, anti-phospho-protein antibodies of selected signaling molecules were used before visualizing with secondary horseradish peroxidase-conjugated antibodies (goat anti-rabbit and goat anti-mouse) from Bio-Rad Laboratories, Inc. (Hercules, CA, USA, #1721011 and #1706515) and an enhanced chemiluminescence reagent (Bio-Rad, #1705060). Representative Western blots from at least 3 independent experiments that resulted in similar outcomes are presented. Total protein staining either in PAA gel with Coomassie R-250 brilliant blue dye (Thermo Scientific, Rockford, IL, USA, #24615) or Pierce Reversible Protein Stain Kit for PVDF Membranes (Thermo Scientific, #24585), served as loading controls.

Primary antibodies used: pT308 Akt (Cell Signaling Technology, #2965), pT202-pY204/pT185-pY187 ERK1/2 (Santa Cruz Biotech, sc-16982-R; Biotechne, AF1018), pGSK3beta (Cell Signaling, #5558), pan-cytokeratin (Santa Cruz Biotech, sc-8018; Thermo Fisher, MA5-12281), vimentin (Sigma, V6389), GAPDH (Abcam, ab9484).

### 2.7 Statistical analysis

The data in cell viability charts are expressed as means ( ± SD) of at least three independent experiments performed in quadruplicate. The data in qRT-PCR charts are expressed as means ( ± SD) of two technical replicates from selected representative samples.

## 3 Results

### 3.1 Characterization of human NSCLC tumor-derived cell lines

**Figure 1:**
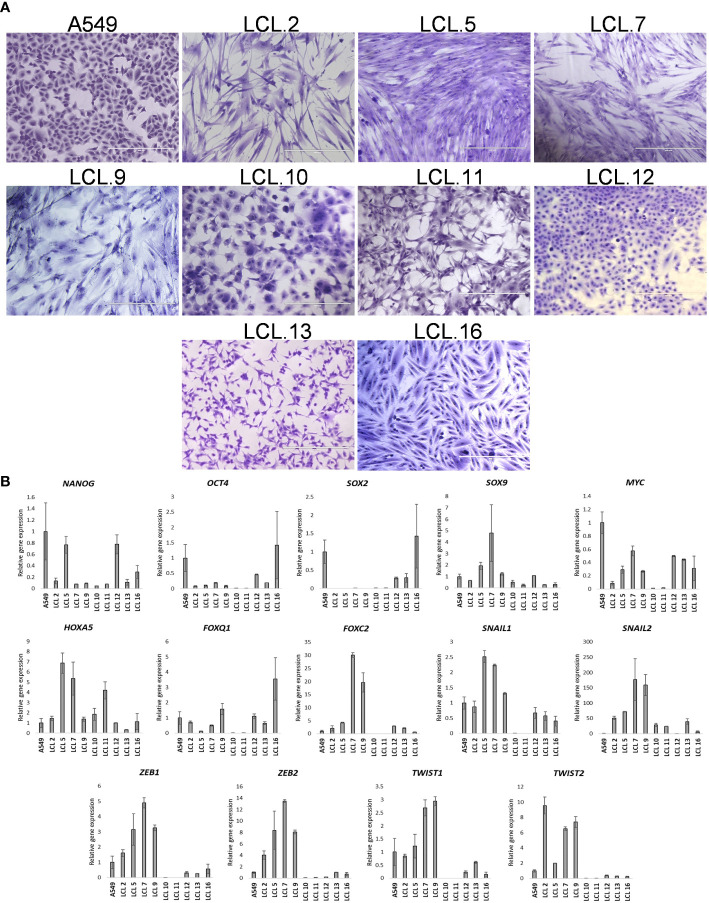
Human NSCLC tumor-derived cell line characterization. **(A)** Morphology of cells from A549 and primary cell lines grown *in vitro* for more than 10 passages. Scale bars = 400 µm. Cells have been stained with crystal violet dye. **(B)** The relative expression of stem cell- and EMT-related transcription factors as determined by RT-qPCR (in respect to *GAPDH*). Data are presented as the mean ± SD (N = 2).

Phenotypically and/or genotypically different primary cell lines, derived from lung cancer (NSCLC) patients’ tumors, passaged over 10 times *in vitro* were used in our studies. The continuous cell line A549 was taken as a basis for comparison. Our previous studies using flow cytometry analysis showed that established human non-small cell lung cancer primary cell lines variously expressed putative lung cancer stem cell surface markers (34). In **Figure 1**, we present a further characterization of these cells in culture.

**Figure 2:**
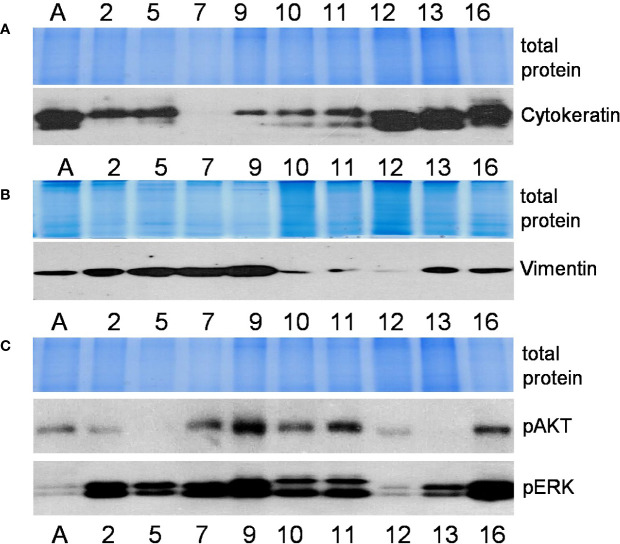
Characterization of cell lines (continued). Relative expression of mesenchymal and epithelial markers cytokeratin **(A)** and vimentin **(B)** (proteins of intermediate filaments family) and activated protein kinases phospho-ERK and phospho-AKT **(C)** as determined by the Western blot method. Representative pictures from more than 3 independent experiments are shown. Coomassie-stained polyacrylamide gels serve as loading controls. Numbers indicate cell lines as in **Figure 1**; the letter A stands for the cell line A549.

Microphotograph images show cells of different morphology, epithelial or mesenchymal phenotypes (**Figure 1A**). Expression studies of core stemness as well as epithelial-mesenchymal transition (EMT)-related transcriptional regulators that are involved in the development and progression of cancer, including lung cancer - NANOG, OCT4, SOX2, SOX9, MYC, HOXA5, FOXO1, FOXC2, SNAIL1, SNAIL2, ZEB1, ZEB2, TWIST1, and TWIST2, revealed that the cells were differently positive for these stemness- and EMT-related transcription factors (**Figure 1B**). Co-expression of proteins belonging to the intermediate filament family, cytokeratin and vimentin, as detected by Western blotting (**Figures 2A, B**), demonstrates cellular states with the features of partial EMT. Subsequent studies were performed to evaluate basal ERK and AKT phosphorylation levels in the acquired cell lines. The proteins of PI3K/Akt and MAPK pathways that regulate cell proliferation and survival, AKT and ERK, are often activated in cancer cells. The data presented in **Figure 2C** show phosphorylated ERK and AKT kinases in cell lines although to varying degrees. Some changes in the cells of the studied cell lines are observed during their *in vitro* cultivation.

### 3.2 Impact of cisplatin and targeted drugs on cell viability and phosphorylation of ERK1/2 and AKT in different lung cancer-derived cell lines

**Figure 3:**
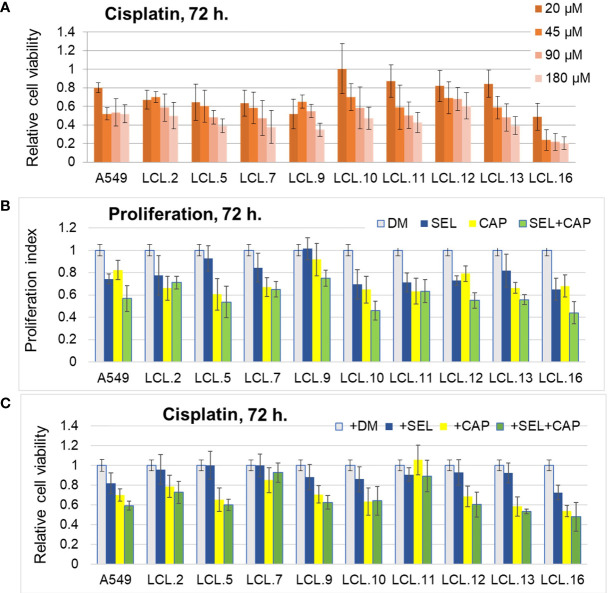
Impact of cisplatin and targeted drugs on cell viability of different lung cancer-derived cell lines. **(A)** Lung cancer-derived cells respond differently to cisplatin. Cell viability was determined by MTT assay and expressed in relation to the viability before the treatment (relative cell viability of initial control =1.0). DM – solvent DMSO. Data are expressed as means ± SD, N = 4. **(B)** Targeted drugs inhibit cell proliferation. Proliferation without inhibitors is normalized to 1.0. **(C)** Lung cancer cells respond differently to combinations of targeted drugs and conventional drug cisplatin. Cell survival without inhibitors is normalized to 1.0. Concentrations of cisplatin that induced 50% cell death after 72 h of treatment were used for each cell line. DM – vehicle control (DMSO), SEL – selumetinib (10 µM), CAP – capivasertib (10 µM).

Further in this study MEK/ERK and AKT kinase-targeted drugs and their combinations with conventional anticancer drugs were tested on lung cancer-derived cell lines. After evaluation of efficacy of most commonly used chemotherapeutics in the clinical treatment of lung cancer (i.e., cisplatin, paclitaxel, and docetaxel) on our model cells, cisplatin was chosen for further viability/cytotoxicity experiments, as well as targeted drugs described in Materials and Methods. *In vitro* drug testing in a series of dose-response experiments demonstrated differential drug sensitivity of the cell lines, as well as unequal response to conventional chemotherapeutic drug cisplatin and targeted drugs, directed at the molecules of ERK and AKT signaling pathways (**Figure 3**). Viability tests presented in **Figure 3A**, showed a nonuniform cisplatin concentration-dependent decrease in cell viability in different lung cancer-derived cell lines as determined after 72 hours of treatment. Intracellular kinase ERK and AKT-targeted drugs resulted in cell proliferation inhibition (**Figure 3B**), but had modest and different (among cell lines) effects on cisplatin-induced cell death when used alone (**Figure 3C**). Meanwhile, the combination of inhibitors of ERK (selumetinib) and AKT (capivasertib) signaling led mostly to an increase in both anti-proliferative and pro-death responses, although again in a cell-line-dependent manner. The results are shown in **Figures 3B, C**.

Although in general cells were quite resistant to the drugs studied (note that the inhibitors of kinases alone did not induce cellular death although they slowed down the proliferation; similarly, the highest concentration of cisplatin did not kill more than 60% (on average) of the cells in 3 days), cell behavior indicated that both ERK and AKT were involved in the regulation of cell proliferation and survival after treatments.

**Figure 4:**
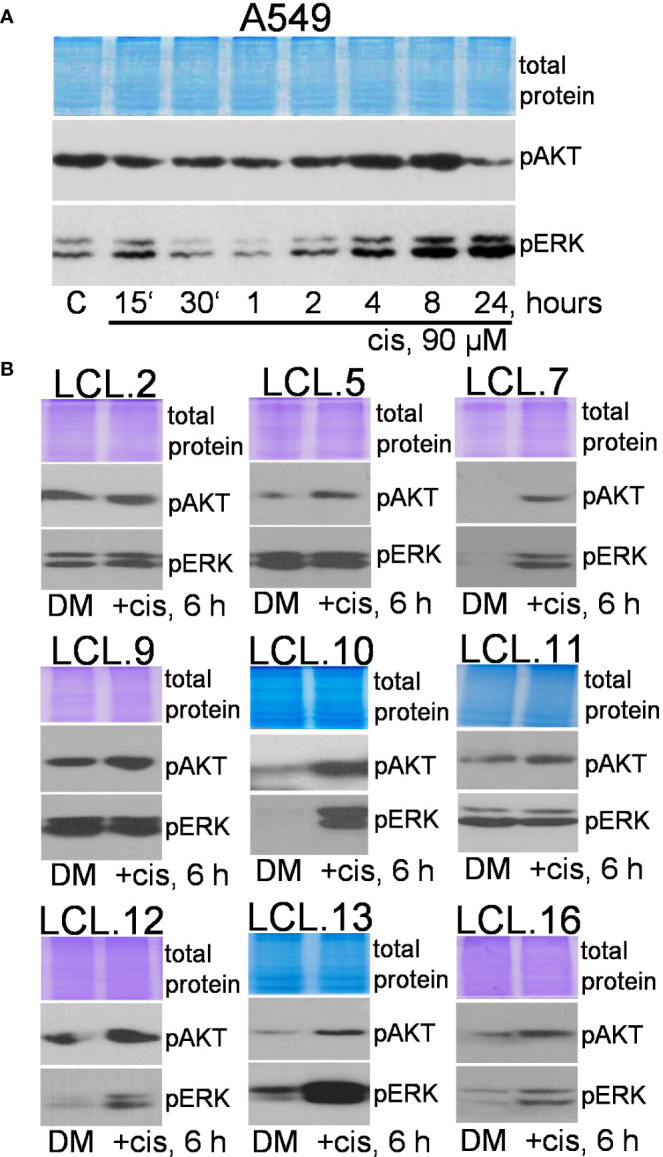
Phosphorylation of ERK1/2 and AKT in lung cancer-derived cell lines after cisplatin treatment. **(A)** The dynamics of AKT and ERK phosphorylation in A549 cells. **(B)** Changes in AKT and ERK phosphorylation in lung tumor-derived cell lines after 6 hours of cisplatin treatment. Representative Western blots are shown. Coomassie-stained protein gels are presented as loading controls. The concentration of cisplatin was 90 µM.

To support this, the changes in the phosphorylation status of these protein kinases were studied after cisplatin treatment. WB analysis showed an increase in the phosphorylation of both kinases. As shown in **Figure 4A**, the treatment of A549 cells with cisplatin (90 µM) resulted in a gradual and persistent increase in phosphorylation of ERK. Less noticeable changes in phosphorylation of AKT were observed. An increase in ERK and AKT phosphorylation, as presented in **Figure 4B**, was also shown in other cell lines in most cases after 6 hours of cellular exposure to cisplatin.

Therefore, in most cases, constitutively phosphorylated ERK1/2 and AKT were further stimulated by chemotherapeutic cisplatin in studied cells, but the level of phosphorylation was cell-, drug-, and concentration-dependent. Expression of total ERK and AKT proteins was not altered by cisplatin (see the **Supplement**).

### 3.3 Modulation of ERK and AKT phosphorylation by targeted drugs

**Figure 5:**
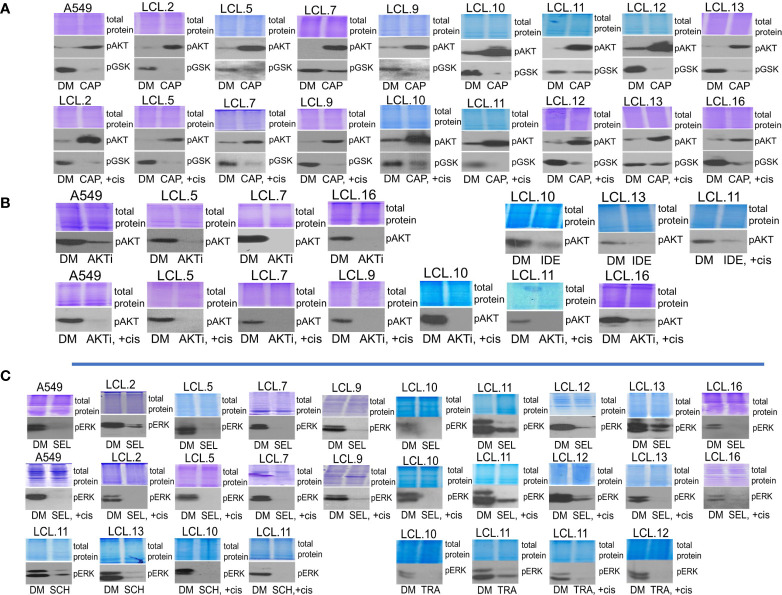
Determining the efficacy of targeted drugs by phosphorylation of their targets. **(A)** Capivasertib, an AKT inhibitor, increases the phosphorylation of AKT itself. Capivasertib efficacy is demonstrated by inhibited phosphorylation of GSK3β, a downstream target of AKT. DM – vehicle control (DMSO), CAP – capivasertib, +cis – cisplatin. **(B)** AKT inhibitor VIII (AKTi) and PI3K inhibitor idelalisib **(IDE)** reduce AKT phosphorylation in control and cisplatin-treated cells. **(C)** MEK/ERK signaling inhibitors suppress ERK1/2 phosphorylation in control and cisplatin-treated cells. SEL – selumetinib, SCH – ERK inhibitor SCH772984, TRA – trametinib. Representative Western blots are shown. Coomassie-stained protein gels are presented as loading controls. 6-hour-long exposures to the drugs were used. The concentration of cisplatin was 90 µM, and that of inhibitors was 10 µM except for trametinib and SCH772984 (1 µM).

Further, the efficacy of the inhibitors used in our study was confirmed by reduced target phosphorylation in all tested cells. We would like to note that the AKT inhibitor capivasertib increased the phosphorylation of AKT itself at position Thr308, but at the same time it inhibited the phosphorylation of AKT downstream target GSK3β (**Figure 5A**), thus confirming the inhibition of kinasic activity of AKT. Data on the increase in AKT phosphorylation upon exposure of cells to the capivasertib are also shown by other authors (35–37). As shown in the **Figure 5B**, preincubation of cells for 30 min with targeted drugs inhibiting AKT signaling (AKT kinase inhibitor VIII; idelalisib, an inhibitor of AKT upstream kinase PI3K), effectively decreased phosphorylation of AKT molecules in control and cisplatin-treated cells. At the same time, inhibitors of ERK signaling (selumetinib and trametinib, inhibitors of ERK upstream kinases MEK1/2, and ERK inhibitor SCH772984) decreased phosphorylation of ERK (**Figure 5C**). Experiments confirmed that the used targeted drugs were effective in our cellular model system.

### 3.4 Negative feedback interactions between MEK/ERK and PI3K/AKT signaling pathways in lung tumor-derived cell lines

Activation of alternative kinases in cells using targeted drugs was investigated as a possible mechanism of resistance to target drugs. The phenomenon of crosstalk between PI3K/AKT and MEK/ERK pathways was analyzed by using inhibitors of molecules of these signaling pathways alone or in combination with cisplatin in a panel of lung cancer-derived primary cell lines.

**Figure 6:**
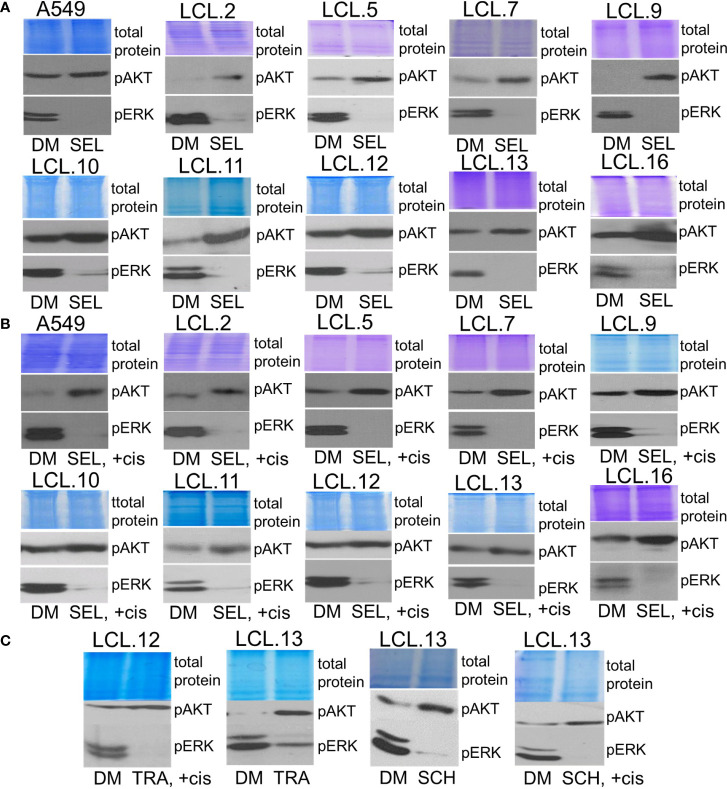
Crosstalk with negative feedback between ERK and AKT signaling pathways in human lung cancer-derived cell lines. **(A, B)** MEK/ERK pathway inhibitor selumetinib enhances AKT phosphorylation in the cell lines tested, control **(A)** and cisplatin-treated cells **(B)**. **(C)** MEK inhibitor trametinib as well as ERK inhibitor SCH772984 increase AKT phosphorylation in control and in cisplatin-treated cells. Representative Western blots are shown. Coomassie-stained protein gels are presented as loading controls. 6-hour-long exposures to the drugs were used. DM – vehicle control (DMSO), SEL – selumetinib (10 µM), +cis – cisplatin (90 µM), TRA – trametinib (1 µM), SCH - SCH772984 (1 µM).

Bearing in mind that *KRAS* status can determine feedback between PI3K/AKT and MEK/ERK pathways (38), in our work we used *KRAS-*mutant A549 cells (a continuous cell line) to compare the phenomenon of activation of alternative kinases with other cell types. We tested cell lines obtained from lung tumors, albeit without genetic subtyping, and found that the phenomenon of crosstalk was common to all cell types studied. As shown in **Figure 6**, treatment of cells with the inhibitors of ERK signaling selumetinib, trametinib, and SCH772984 increased AKT phosphorylation both in control and cisplatin-treated cells.

**Figure 7:**
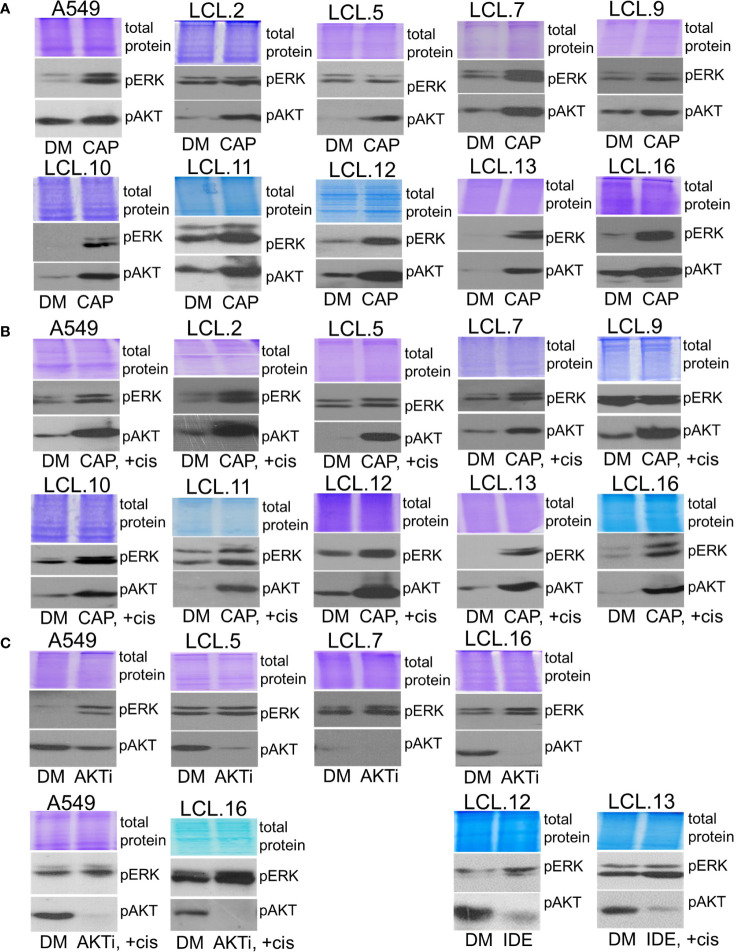
Crosstalk between ERK and AKT (continued). **(A, B)** AKT inhibitor capivasertib enhances ERK1/2 phosphorylation in control **(A)** and cisplatin-treated cells **(B)**. **(C)** AKT inhibitor VIII enhances ERK1/2 phosphorylation in the cell lines tested, both control and cisplatin-treated cells. Similarly, PI3K inhibitor idelalisib increases ERK phosphorylation in human lung-derived cell lines. Representative Western blots are shown. Coomassie-stained protein gels are presented as loading controls. 6-hour-long exposures to the drugs were used. DM – vehicle control (DMSO), CAP – capivasertib (10 µM), +cis – cisplatin (90 µM), AKTi – AKT inhibitor VIII (10 µM), IDE – idelalisib (10 µM).

Conversely, inhibition of AKT signaling with capivasertib, idelalisib, and AKT inhibitor VIII increased ERK phosphorylation in all cell lines examined, again both in control and cisplatin-treated (**Figure 7**). Thus, compensatory feedback loops between the two signaling pathways have been confirmed in the lung-derived cells: inhibition of ERK induced activation of AKT and vice versa: inhibition of AKT led to activation of ERK. It should also be mentioned that the feedback phenomenon between kinases has also been observed in our previous work on cells of non-human origin, namely, rabbit muscle-derived stem cells (data not presented). In addition, the inhibitors had no (or negligible) impact on total amounts of EKR2 and pan-AKT protein levels as determined by using Western blot method (see the **Supplement**).

Therefore, the phenomenon of crosstalk between kinases ERK1/2 and AKT was demonstrated with all different targeted drugs (kinase inhibitors) used in our study and was evidenced in all cell lines tested, regardless of genotype and phenotype differences.

### 3.5 Possible role of extracellular contacts and chemotherapeutic drug concentration in the regulation of AKT-ERK crosstalk

**Figure 8:**
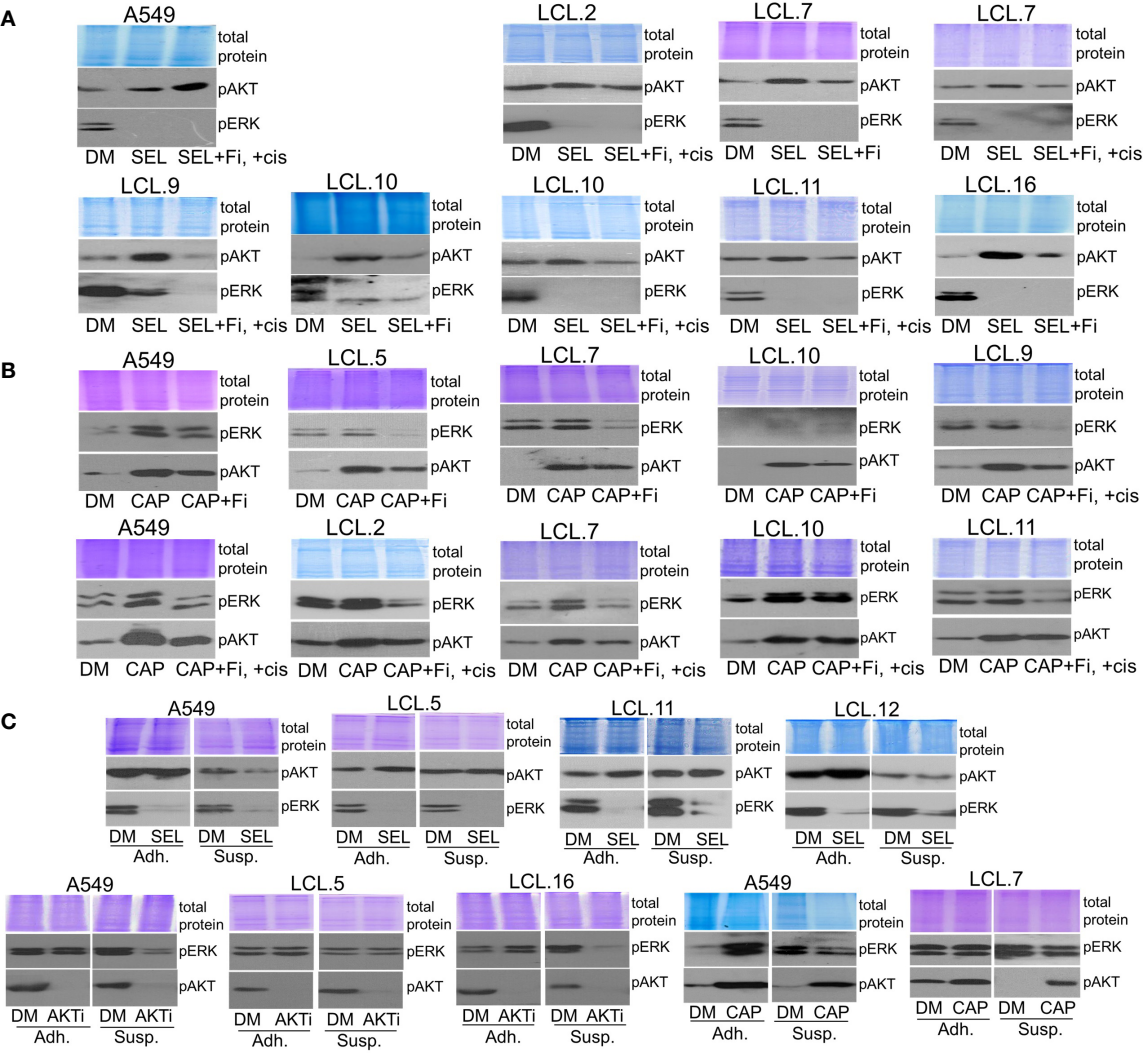
Dependence of the interplay between ERK and AKT signaling pathways on extracellular contacts. **(A)** Focal adhesion kinase inhibitor PF573228 (+Fi; 10 µM) attenuates the MEK kinase inhibitor selumetinib-induced increase of AKT phosphorylation in control and cisplatin-treated lung cancer-derived cells, except A549 cells. **(B)** PF573228 prevents the AKT inhibitor capivasertib-induced increase in ERK phosphorylation in control and cisplatin-treated lung cancer-derived cells. **(C)** Cells grown in suspension (Susp) under agitation, in contrast to adherent cells (Adh), do not show alternative kinase activation after the treatment with inhibitors. Representative Western blots are shown. Coomassie-stained protein gels are presented as loading controls. 6-hour-long exposures to the drugs were used. DM – vehicle control (DMSO), SEL – selumetinib (10 µM), +cis – cisplatin (90 µM), CAP – capivasertib (10 µM), AKTi – AKT inhibitor VIII (10 µM).

Next, to better understand the interconnectivity between kinases, in this study, we investigated possible regulatory mechanisms of identified negative feedback loops and crosstalk phenomenon by inhibiting the molecules of PI3K/AKT and MEK/ERK signaling pathways in tested cells. Feedback mechanisms concerning extracellular contacts and the extent of treatment (drug dosing) were investigated. Our results presented in **Figures 1**–**7** were performed with cells adhering to the extracellular substrate, and we further sought to elucidate how kinase interactions might be regulated in circulating cancer cells. In this study, still using adherent cell cultures, we observed that an inhibitor of focal adhesion kinase PF573228 attenuated AKT phosphorylation which was induced by MEK inhibitor selumetinib (**Figure 8A**). Similarly, AKT inhibitor capivasertib-induced increase in ERK phosphorylation was diminished by PF573228 in control and cisplatin-treated lung cancer-derived cells (**Figure 8B**). This suggested a possible role of cellular interactions with the substrate, in regulating the crosstalk between the signaling pathways.

It should be mentioned that we failed to obtain a decrease in AKT phosphorylation in A549 cells exposed to PF573228 and selumetinib simultaneously (**Figure 8A**). These data are consistent with our previously demonstrated role of FAK inhibition on ERK kinase in A549 cells: FAK inhibitor promoted ERK phosphorylation in A549 cells. Likewise, abnormal activation of ERK1/2 in lung cancer A549 cells, in contrast to downregulation in other cell lines studied, was determined in an anchorage-independent state (34).

Lastly, to mimic the anchorage-independent state of circulating cancer cells, we incubated single cells of randomly selected lines in suspension without any cell-cell contacts as described in the Methods section and treated them with drugs.

As we can see in **Figure 8C**, the effect of inhibitors on the activation of alternative kinases, showing crosstalk between kinases, was attenuated in cells in suspension. This phenomenon has been confirmed for selumetinib, capivasertib and AKT inhibitor VIII. No increase in AKT or ERK phosphorylation following ERK or AKT inhibition, respectively, was detected in the non-adherent cell model. Thus, the loss of extracellular contacts may eliminate kinase interactions.

**Figure 9:**
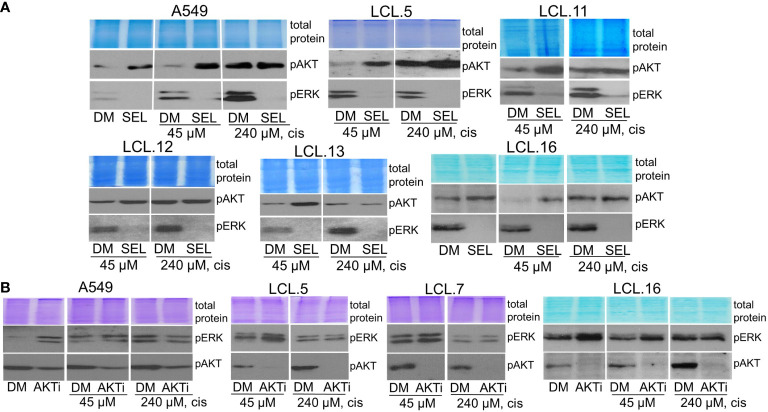
ERK and AKT crosstalk might be signal strength-dependent. Cells pretreated with a high concentration of cisplatin (240 µM) do not show alternative kinase AKT activation in response to ERK inhibition by selumetinib **(A)**, nor ERK activation in response to AKT inhibitor VIII, contrary to untreated or low cisplatin concentration (45 µM)-treated cells **(B)**. Representative Western blots are shown. Coomassie-stained protein gels are presented as loading controls. 6-hour-long exposures to the drugs were used. DM – vehicle control (DMSO), SEL – selumetinib (10 µM), cis - cisplatin, AKTi – AKT inhibitor VIII (10 µM).

In addition, in this study, we found that interaction between the kinases may be dependent on the strength of the chemotherapeutic stimulus: no crosstalk between AKT and ERK1/2 was observed in cells exposed to a high concentration (240 µM) of cisplatin (**Figure 9**). The latter finding might support the idea of high-concentration intra-tumoral chemotherapy (39).

Based on the obtained data, it can be stated that the intensity of drug-induced signaling as well as signals induced by extracellular contacts may be involved in the interplay between kinases.

## 4 Discussion

Although cancer is caused by genetic mutations, there is growing evidence that non-genetic mechanisms lead to drug resistance.

There is no doubt that identification of genetic tumor signatures is of great importance for cancer treatment, however, now it is well known that non-genetic modulation of the cellular state can strongly influence drug responses, conferring resistance to both conventional cytotoxic and targeted drugs.

A variety of non-genetic mechanisms of cell resistance influencing drug responses have been described in many cancers. Usually, phenotype switching, cell dynamic state, and extracellular contacts, along with the identification of molecular mechanisms, are assessed during the evaluation of drug effectiveness (1). We rely on the opinion that cell state-specific therapy is one of the essential approaches in modern cancer treatment (29), where cell fate may be determined by the activity of MEK/ERK and PI3K/AKT signaling pathways, influenced by cell-specific intrinsic and extrinsic sources. The efficacy of targeted therapies against these signaling pathways, however, is limited due to the acquisition of non-genetic adaptive resistance by tumor cells. Therefore, the combination of specific signaling pathway-targeted drugs provides new treatment strategies (4, 31, 40).

The interaction between these signaling pathways has been shown for various and different cancer cell models (35, 38, 41–43). However, the prevailing view is that feedback between the AKT and ERK pathways is cell line-specific and depends on specific oncogenic mutations in cancer cells. In our work, on the contrary, the crosstalking of both signaling pathways was shown to be common to all cell types (at least to the ones we have examined) – inhibition of the PI3K/AKT pathway led to activation of the MAP kinase ERK and, conversely, inhibition of the MEK/ERK pathway increased phosphorylated AKT levels both in control cells, and in cells exposed to the chemotherapeutic drug cisplatin.

Given that non-genetic modulation of cell state can strongly affect cell signaling, including interactions between signaling pathways, and that cell state dictates drug sensitivity, we studied the interaction between AKT and ERK signaling molecules in cells that lose contact with the substrate. It may be that circulating tumor cells, key players in metastatic dissemination, may respond differently to anticancer drugs than cells adherent to the extracellular substrate (34). Activation of MAP and AKT kinase cascades is known to involve regulatory events initiated by extracellular contacts through activation of integrins and downstream focal adhesion kinase FAK. Integrins and their associated regulatory signaling pathways regulate diverse cellular functions and are involved in various human cancers (44, 45). Downstream of the integrin and growth factor receptors, FAK activates multiple targets, including AKT. We have previously shown that FAK is involved in the transmission of integrin-induced signaling to ERK and AKT in muscle stem cells and lung cancer cells (34).

Indeed, studies using a focal adhesion kinase inhibitor, as well as comparing substrate-attached cells with cells in suspension, have shown that loss of extracellular contacts can abrogate compensatory interactions between these signaling pathways.

Thus, we have demonstrated the dependence of interactions between kinases on the cellular state. Studies indicate that circulating cells may respond differently to anticancer drugs than cells that encounter other cells or the extracellular substrate. It follows that understanding the signaling events underlying the response of circulating tumor cells to anticancer drugs may lead to new approaches to overcoming the resistance of metastasizing cells to therapy, which unfortunately is a major shortcoming in cancer treatment.

In addition, our work demonstrated that signaling crosstalk was dependent on the relative strength of the inducer, with no cross-inhibition of the opposite kinase observed at high concentrations of cisplatin in different cells.

Feedback interactions between signaling molecules resulting in cross-inhibition of parallel kinases are one of the main problems in molecular targeted therapy for many types of cancer. From that point of view, finding new clinically relevant drug combinations for a variety of different disease forms and states is a primary challenge. The reciprocal cross-inhibition of the RAS-MAPK and PI3K-mTORC1 pathways (when inhibition of one pathway releases signaling through the other pathway) has been studied, discussed, and reviewed by various authors and explained by the specific interaction mechanisms of signaling molecules in different cancer model systems. Activation of this kind of feedback mechanism is recognized as a way to protect cells from drug-induced death (43, 46, 47).

Hence, it can be seen that positive and negative feedback loops and crosstalk have been documented to be dynamic and complex and the relative importance of each interaction may vary across different tissue or tumor types and can also depend on the relative strengths of signaling.

Multiple levels of crosstalk between the PI3K/AKT and RAS/MAPK exist. The pathways could be interconnected with many points of intersection at different sites and phases of signal transduction, crosstalk, and feedback loops. Both pathways can either activate or inhibit each other. Cross-inhibition and cross-activation, as well as pathway convergence, sometimes with the capability of phosphorylating the same motif within the same protein, have been described. Even more, crosstalk is context-dependent and may depend on the dosage of growth factors or other stimuli. In that sense, modeling approaches exploiting the key feedback loops were used to generate new potential mechanisms that could explain the cellular response (43, 46–48).

Various causes and mechanisms of interactions between these two key signaling pathways in different cellular systems have been described in the literature (49). The works indicate a dependence of interplay between signaling pathways on the mutational status of the cell. For example, studying the effects of RAF inhibitors on PI3K/AKT signaling has shown that AKT phosphorylation in cells may change in the opposite manner depending on mutations in two important oncogenes, *KRAS* and *BRAF* (42). Other authors, by using a representative panel of cancer cells of different origins (breast, lung, prostate, oesophageal, and colorectal cell lines) with known genetic background, highlighted that compensatory mechanisms and feedback between the PI3K/AKT and RAS/MEK/ERK pathways are cell line-specific and that oncogenic driver mutations have a decisive role. It was demonstrated that feedback interaction between these two pathways only occurs in *KRAS-*mutant and *cMET* amplified cells but not in the isogenic wild-type cells through a mechanism that may involve inhibition of a specific endogenous phosphatase activity. In addition, inhibition of protein phosphatases in wild-type cells could enable the feedback loop in them (38). The findings led to the conclusion that genetic subtyping of primary tumors for *KRAS* mutation or *cMET* amplification would be valuable for predicting tumor response to the pathway inhibition. Furthermore, Turke et al. (50) described a mechanism, by which MEK supposedly inhibits the ERBB receptors by phosphorylating a certain amino acid, mutations of which in *EGFR* or *HER2* prevented ERBB3/PI3K/AKT crosstalk with MAPK signaling following treatment with MEK inhibitors.

Elsewhere it is explained, that AKT negatively regulates ERK activation during strong IGF1 stimulation, by phosphorylating inhibitory sites at the N-terminus of RAF (51). ERK inhibition by AKT involves EGF-induced ERK phosphorylation of GAB1, which inhibits GAB1-mediated recruitment of PI3K to EGF receptors (52–54). Studies of the mechanism of interplay between the pathways revealed that AKT suppresses ERK activity through direct association with RAF, causing its inactivation *via* phosphorylation of a negative regulatory residue Ser259 (21, 51). Other possible mechanisms involved in the compensatory interaction between the investigated pathways are HER2 phosphorylation at Thr701 and the interaction between HER2/EGFR and clathrin binding (55), as well as adaptive changes in the MAPK scaffolding proteins (KSR-1, GEF-H1) and receptor tyrosine kinases (MET, EGFR), leading to enhanced PI3K/AKT signaling in *KRAS*-mutant lung adenocarcinoma cell lines as revealed by mass spectrometry-based phosphoproteomics (33).

Research of the adaptive resistance to targeted therapies directed at the RAS-ERK signaling pathway has demonstrated the role of integrin, FAK, and an intact actin cytoskeleton in a subset of metastatic triple-negative breast cancer cells. Analyzing the crosstalk mechanisms of the RAS-ERK and PI3K/AKT signaling, the authors found that activation of PI3K/AKT after RAS-ERK pathway inhibition required β1 integrin, myosin light chain kinase (MLCK), and myosin IIA. However, activation of the PI3K/AKT pathway was independent of EGFR signaling (45). In addition, it was indicated that integrin-linked kinase (ILK) was required to mediate feedback activation of the PI3K/AKT pathway following MEK suppression in glioblastoma cells (56).

Metabolic pathways, such as the mevalonate pathway, can also be involved in the regulation of AKT activation induced by MEK inhibitor treatment. The authors propose that combinatorial treatment of MEK inhibitors with antilipidemic drugs statins may be a promising therapeutic strategy to sensitize cancer cells to apoptosis (57).

The phenomenon of a negative feedback loop, when inhibition of one pathway can lead to compensatory activation of the other pathway, is demonstrated in various types of cancer cells with certain types of molecular alterations. Herewith, the efficacy of the combined use of various kinds of MEK and PI3K inhibitors is increasingly being tested in various types of cancer by using laboratory-based models and in clinical trials of selected cancers (43, 58).

The authors propose that the network of crosstalk between the PI3K/AKT and RAS/MEK/ERK pathways may vary across different tissue or tumor types. For example, RAS-RAF-MEK-ERK and PI3K/AKT signaling pathways can crosstalk in the human breast cancer cell line MCF-7 depending on the cellular background or stage of differentiation. The type of ligands, ligand concentration/signaling intensity, and time courses may contribute to crosstalk between these signaling pathways (53). To assess whether crosstalk is conserved in cancer cells of different origins, the effect of combining various inhibitors was examined in a wide variety of cell types (38, 41–44, 59). In lung cancer it is shown that mechanisms of the acquired resistance to receptor tyrosine kinase‐targeted therapy (TKI) involve secondary *EGFR* T790M mutation, *MET* amplification, activation of the mesenchymal‐epithelial transition factor/hepatocyte growth factor axis, induction of epithelial‐to‐mesenchymal transition, acquisition of stem cell properties, and transformation from NSCLC into small cell lung cancer, etc. Compensatory activation between central oncogenic pathways could be another key determinant of drug resistance as well as another target. A feedback loop between the MEK/ERK and PI3K/AKT pathways has been demonstrated in several experimentally established EGFR-TKI-resistant NSCLC cell lines, leading to resistance to treatment with a single inhibitor of either of these signaling pathways. Authors suggest the dual inhibition of MEK plus PI3K pathways with selected inhibitors as a potential therapeutic strategy for NSCLC resistant to EGFR‐TKI (60).

Current evidence clearly indicates that the MAPK and AKT signaling pathways are the most prominent clinically used targets in targeted cancer therapy (5, 49). Various pharmacological agents targeting the RAS/MEK/ERK and PI3K/AKT pathways have been developed and are being investigated in preclinical and clinical trials; unfortunately, limited antitumor activity mainly due to compensatory activation of these key intracellular signaling pathways is one of the major obstacles to their use.

MAPK inhibitors are being extensively evaluated in non-small cell lung cancer patients (7). Studies conducted in Canada have shown that selumetinib, at the determined dose, can be safely combined with paclitaxel and carboplatin or pemetrexed and cisplatin in patients with advanced or metastatic NSCLC (61). However, there is disappointing data from the phase III trial in which selumetinib+docetaxel in patients with advanced *KRAS-*mutant lung cancer did not improve overall survival (62). Similarly, selumetinib+gemcitabine regimens have not been tolerated during the SELECT-3 clinical trial (63). In summary, it should be said that studies utilizing this drug as monotherapy did not confirm its efficacy for NSCLC (64, 65).

The PI3K/AKT/mTOR pathway is also promising target for the treatment of solid cancers. The AKT signaling is constitutively active in non-small cell lung cancer cells (66, 67). More than 50 compounds targeting key components of the PI3K/AKT/mTOR signaling network are in development. Many of them have been tested in clinical trials involving patients with a range of different cancers as this pathway is dysregulated almost in all human cancers, including breast cancer, colorectal cancer, lung and hematologic malignancies, etc. Although the mTOR inhibitors temsirolimus and everolimus and the PI3K inhibitors idelalisib and copanlisib have been approved by the FDA for clinical use in the treatment of some different cancers, clinical data indicated that the use of single-agent PI3K pathway inhibitors achieved modest responses and was unlikely to be a curative therapy for diverse cancers (68–71).

Studies of cross-talks between PI3K signaling and other pathways are thought will guide future combination strategies of using clinically relevant inhibitors in various tumor types (72, 73). Overall, the available evidence suggests that drug resistance (intrinsic or acquired) to such targeted therapies results at least in part from negative feedback interactions between these kinases, combined with tumor heterogeneity, cellular state, mutational background, etc. Therefore, combinations of inhibitors are preferred for cancer treatment (rather than monotherapy) that target different kinases alone or in combination with chemotherapy and/or immunotherapy (47, 60, 74).

A growing number of clinical trials [24 clinical trials till 2018, Table 4 in (75)] are currently underway to evaluate the combination of PI3K and MEK inhibitors in various cancers with specific types of molecular alterations (e.g. RAS/RAF/MEK/ERK pathway activation), namely malignant melanoma with *BRAF* or *NRAS* mutations and colorectal, ovarian, pancreatic, and basal-like breast cancers, etc., with various response results (30, 76–79). Also, combination therapy using MEK and PI3K inhibitors has been proposed as a potent treatment strategy for NSCLC with acquired resistance to EGFR-TKIs (60).

Based on our studies, it can be concluded that the usefulness of a combination of kinase inhibitors should be evaluated for each cancer subtype based on the pattern of MEK/ERK and PI3K/AKT signaling. It appears that cancer cells entering the bloodstream, when compared to the cells within a solid tumor, should respond differently to targeted drugs due to differences in regulation. Further studies are needed to be directed at a more detailed understanding of the molecular mechanisms governing the feedback regulation of protein kinases ERK and AKT in different cellular states and at different inducer concentrations.

## Data availability statement

The original contributions presented in the study are included in the article/**Supplementary Material**. Further inquiries can be directed to the corresponding author.

## Author contributions

AS: Investigation, formal analysis, visualization, writing - review & editing. MS: Investigation, formal analysis. AV: Investigation, formal analysis. AI: Investigation, writing - review & editing. NK: Investigation. AK: Conceptualization, funding acquisition, methodology, resources, investigation, writing - original draft, supervision. All authors contributed to the article and approved the submitted version.
