# Supplementary material for: Crosstalk between protein kinases AKT and ERK1/2 in human lung tumor-derived cell models
Source: Front Oncol. 2023 Jan 4;12:1045521. doi: 10.3389/fonc.2022.1045521 (PMC9848735; doi:10.3389/fonc.2022.1045521)
Supplement: Supplementary file 1 [file DataSheet_1.pdf]

# Supplement

To the Article: ,,Crosstalk between protein kinases AKT and ERK1/2 in human lung tumor-derived cell models"

by Aurimas Stulpinas, Matas Sereika, Aida Vitkeviciene, Ausra Imbrasaite, Natalija Krestnikova, Audrone V. Kalvelyte*

## 1. Exposure to cisplatin does not alter total AKT or ERK2 protein levels in lung tumor-derived cell lines

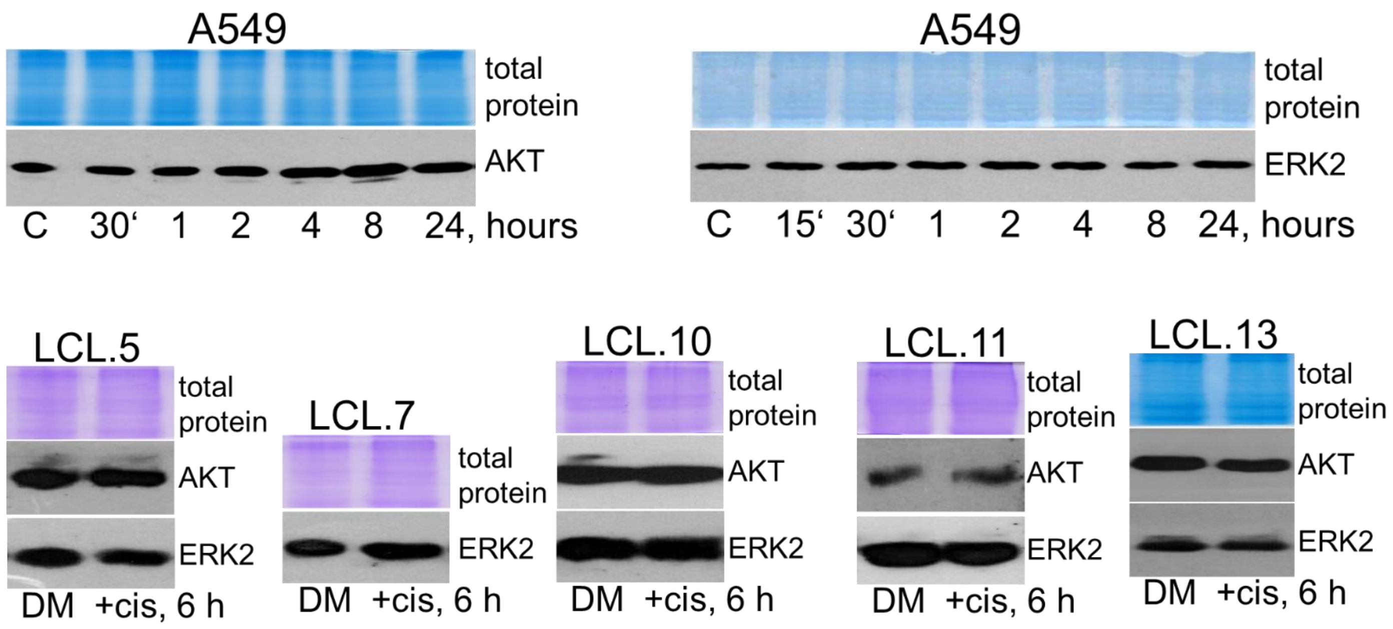


C – control/untreated cells; DM – solvent control (DMSO), +cis – 90 µM concentration of cisplatin. Western blot antibodies: anti-pan-AKT Invitrogen 44-609G; anti-ERK2 SantaCruz sc-1647. The treatments are as described in the main Article.

2. Inhibition of kinase ERK does not affect AKT protein expression in lung tumor-derived cell lines, control cells and after exposure to cisplatin, in contrast to AKT phosphorylation (Fig. 6 in the main Article)

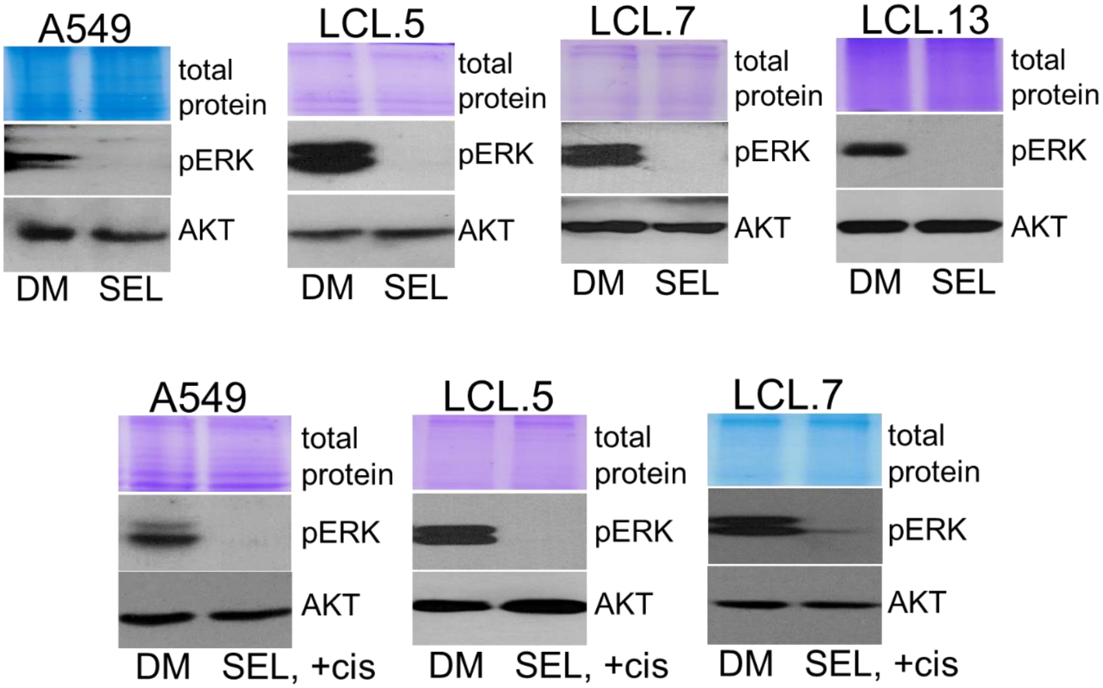


DM – solvent control (DMSO), SEL – selumetinib (10 µM), inhibitor of ERK's upstream kinase MEK; +cis – 90 µM concentration of cisplatin. The treatments are as described in the main Article.

## 3. Inhibition of kinase AKT does not affect ERK2 protein expression in lung tumor-derived cell lines, control or cisplatin-treated, in contrast to ERK phosphorylation (Fig. 7 in the main Article)

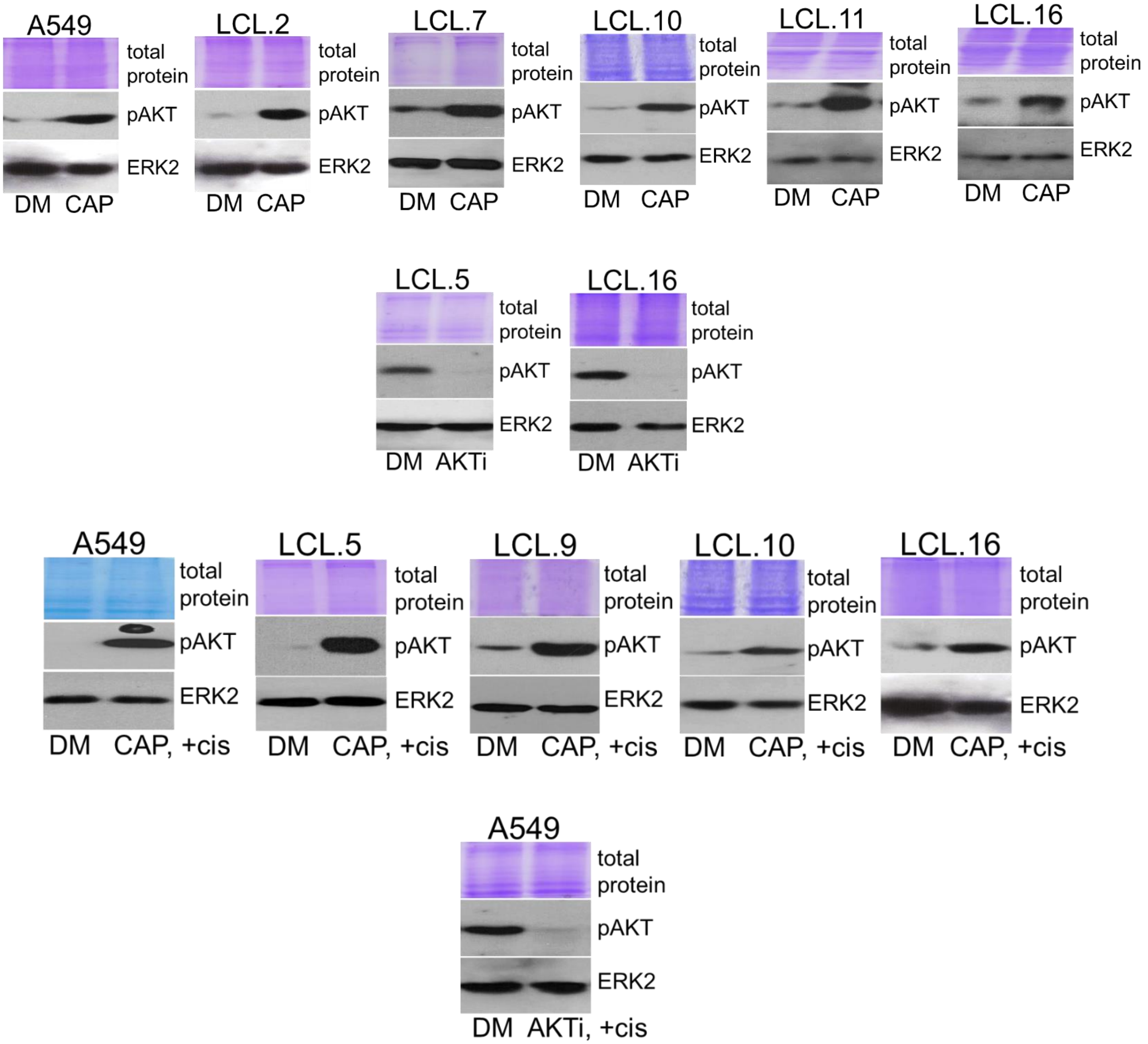


DM – solvent control (DMSO), CAP – capivasertib (10 µM), inhibitor of AKT kinasic activity (although capivasertib increases phosphorylation of AKT at Thr308); AKTi – AKT inhibitor VIII (10 µM), +cis – 90 µM concentration of cisplatin. The treatments are as described in the main Article.

4. FAK inhibitor PF573228 does not affect expression of ERK2 protein in AKT inhibitor capivasertib-treated cells in contrast to decrease of ERK phosphorylation (Fig. 8B in the main Article)

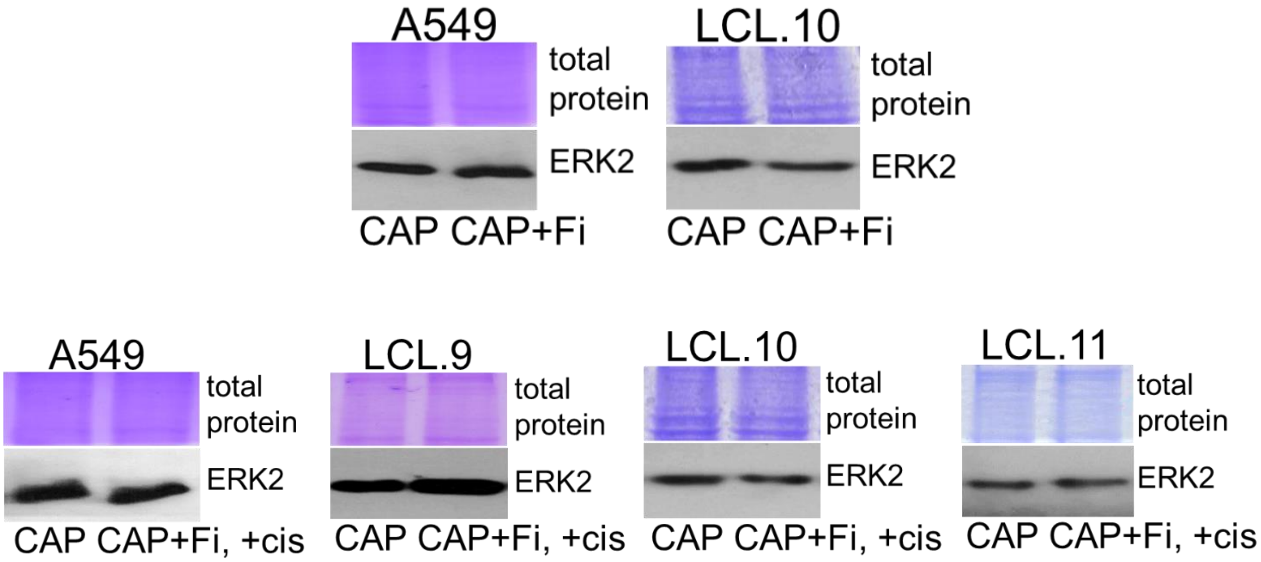


CAP – capivasertib (10 µM), Fi – FAK inhibitor PF573228 (10 µM), +cis – 90 µM concentration of cisplatin.

## 5. Inhibition of either ERK or AKT does not affect protein expression of the alternative kinase in lung tumor-derived cells grown in suspension

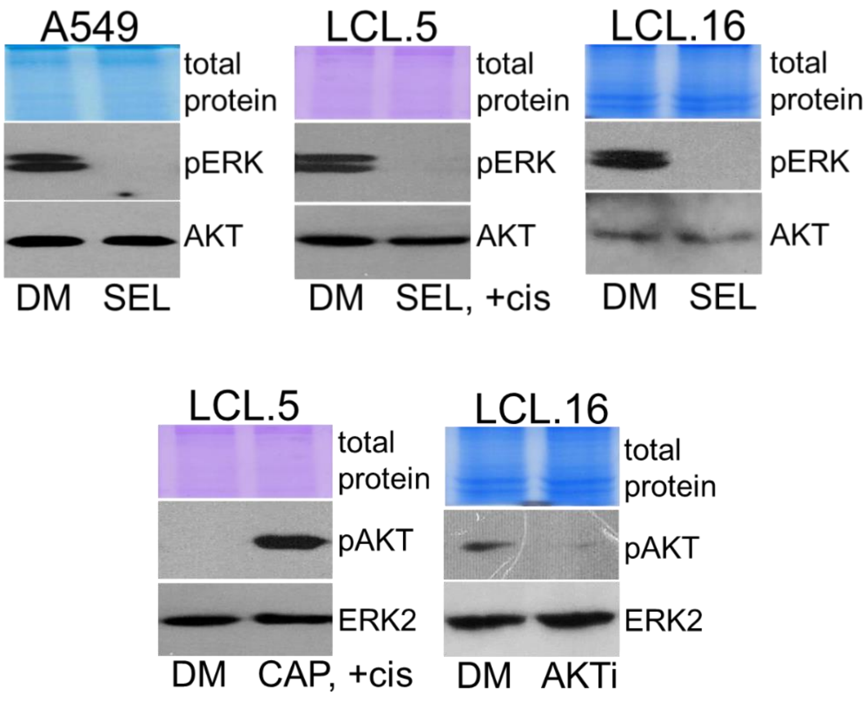


DM – solvent control (DMSO), SEL – selumetinib (10 µM), +cis – 90 µM concentration of cisplatin; CAP – capivasertib (10 µM), AKTi – AKT inhibitor VIII (10 µM). Experimental design is as described in the main Article, except the treatment with cisplatin (LCL.5).

## 6. AKT protein expression does not change after exposure to ERK pathway inhibitor selumetinib at high concentrations of cisplatin

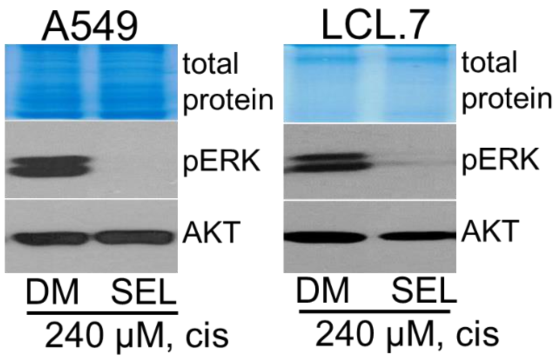


DM – solvent control (DMSO), SEL – selumetinib (10 µM). Six-hour long exposure to cisplatin was used.
